# Predicting the developmental toxicity of 8-methyl-benzo[a]pyrene (BaP) by physiologically based kinetic (PBK) modeling-facilitated reverse dosimetry and read-across from BaP

**DOI:** 10.1007/s00204-025-04115-y

**Published:** 2025-07-03

**Authors:** Danlei Wang, Jing Fang, Miaoying Shi, Lenny Kamelia, Ivonne M. C. M. Rietjens, Peter J. Boogaard

**Affiliations:** 1 Division of Toxicology, Wageningen University and Research, Stippeneng 4, 6708 WE Wageningen, The Netherlands; 2 NHC Key Laboratory of Food Safety Risk Assessment, China National Center for Food Safety Risk Assessment, Beijing, 100021 China; 3 Shell Global Solutions International B.V., 2596HR The Hague, The Netherlands; 4 Member of Concawe, Brussels, Belgium

**Keywords:** PBK modeling, Read-across, Developmental toxicity, Alkyl substituted PAH, 8-Methyl-benzo[a]pyrene

## Abstract

The present study predicts an in vivo dose-response curve for developmental toxicity of 8-methyl-benzo[a]pyrene (8-MBaP) in rats using PBK model-facilitated reverse dosimetry and read-across from a previously developed model for BaP. As for BaP, in vitro developmental toxicity of 8-MBaP in the mouse embryonic stem cell test (mEST) was shown to require bioactivation to its 3-hydroxy metabolite (3-OH-8-MBaP). To facilitate extrapolation of the in vitro observed developmental toxicity of 3-OH-8-MBaP to an in vivo dose–response curve for 8-MBaP, a PBK model for 8-MBaP was built based on the previously developed PBK model for BaP. The PBK models for BaP and 8-MBaP were validated based on (i) comparison of predicted blood concentrations of BaP or 8-MaP to blood concentrations in rats dosed with BaP or 8-MBaP in the present study and (ii) literature-reported blood concentrations of BaP and its metabolite 3-OH-BaP in rats upon dosing with BaP. The predicted half maximal effect dose (ED_50_) for developmental toxicity of 8-MBaP that was derived from the predicted in vivo dose–response curve was 1.3-fold lower than that of BaP for which the predicted value was in line with the reported in vivo ED_50_. The present study illustrates how a new approach methodology (NAM) can be applied to predict developmental toxicity of a methyl substituted PAH, by PBK modeling facilitated read-across from a non-substituted PAH with available in vivo data. The method also elucidates how a methyl substituent could affect the kinetics and subsequent developmental toxicity of a non-substituted PAH.

**Supplementary Information:**

The online version contains supplementary material available at 10.1007/s00204-025-04115-y.

## Introduction

Petroleum substances on the EU market are required to be evaluated for their effect on prenatal developmental toxicity in accordance with the REACH (Registration, Evaluation, Authorization, and Restriction of Chemicals) regulation (EC) 1907/2006 (EC 2006; ECHA 2009). Given the large number of experimental animals potentially needed for safety testing of chemical substances according to the current OECD 414 guideline (OECD 2018), the use of new approach methodologies (NAMs) for the hazard assessment of chemical substances is promoted and supported (ECHA 2014). The development and application of alternative testing strategies for assessing the developmental toxicity of highly complex petroleum substances are highly relevant and important in view of next generation risk assessment (NGRA).

**Fig. 1:**
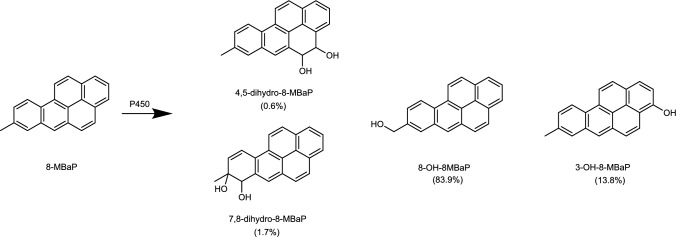
Metabolic profile of 8-MBaP in incubations with rat liver microsomes (Wang et al. 2022). The percentage values shown in brackets were calculated from the concentration of the metabolite divided by the total concentration of metabolites formed in the microsomal incubations

Developmental toxicity, as observed with some petroleum substances, has been associated with the presence of certain polycyclic aromatic compounds (PACs), including polycyclic aromatic hydrocarbons (PAHs), in these products (Dalbey et al. 2014; Feuston et al. 1996, 1997; Kamelia et al. 2017; Murray et al. 2013). The hazardous aromatic constituents in petroleum-derived products comprise 3- to 7-ring PAHs that may be unsubstituted or lowly alkylated (Carrillo et al. 2022). Limited studies on developmental toxicity of the 3- to 7-ring PAHs have been reported in experimental animals. The offspring of pregnant rats exposed to benzo[a]pyrene (BaP), a 5-ring PAH, was affected with regard to embryo lethality, fetal body weight, and incidence of resorptions (Archibong et al. 2002; Bui et al. 1986). Developmental toxicity of BaP was shown in the mouse embryonic stem cell test (mEST) to require metabolic activation to its major metabolite 3-hydroxy-benzo[a]pyrene (3-OHBaP) (Kamelia et al. 2020). Recent findings in the zebrafish embryotoxicity test (ZET) showed that methylated PAHs cause developmental retardation in zebrafish embryos, comparable to the effects induced by their unsubstituted parent PAHs, albeit with different potency. The most potent PAH was 8-methyl-benzo[a]pyrene (8-MBaP) with a BMC_20_ (benchmark concentration causing 20% extra effect above background) value that was ten times lower than that of BaP (Fang et al. 2022). 3-Hydroxy-8-methyl-BaP (3-OH-8-MBaP) was the major oxidative aromatic ring metabolite of 8-MBaP in both rat liver microsomal and S9 incubations (Wang et al. 2022) (Fig. 1). Thus, it is hypothesized that 8-MBaP follows a similar bioactivation pathway as BaP with regard to the induction of developmental toxicity.

Our recent study supported an in vitro–in silico approach to predict the in vivo developmental toxicity of BaP in rats using physiologically based kinetic (PBK) modeling-facilitated reverse dosimetry (Wang et al. 2021). The predicted blood concentrations of BaP and 3-OHBaP were sufficiently validated by comparison to results from toxicokinetic studies in rats (Marie et al. 2010; Moreau and Bouchard 2015). The prediction of the developmental toxicity of BaP upon oral exposure was based on the concentration–response curves of 3-OHBaP obtained in the mEST, and the predicted in vivo dose–response curves for developmental toxicity of BaP obtained by PBK modeling-based quantitative in vitro to in vivo extrapolation (QIVIVE) of these in vitro data that matched the literature-available dose–response curves from in vivo developmental toxicity studies (Archibong et al. 2002; Bui et al. 1986), thereby validating the alternative testing strategy. The aim of the present study was to predict the developmental toxicity of 8-MBaP using PBK modeling-facilitated reverse dosimetry and read-across from BaP based on the same in vitro–in silico approach previously shown successful for BaP (Wang et al. 2021). The PBK model-based predictions for blood concentrations of 8-MBaP or BaP were validated with data from an in vivo toxicokinetic study in rats dosed (intravenously) with either 8-MBaP or BaP and by comparison of predictions made to kinetic data reported in the literature for blood concentrations of BaP and its metabolite 3-OH-BaP in rats upon dosing BaP. The results obtained will elucidate the potential for further use of read-across and NAMs in the hazard and risk assessment of alkyl substituted PAHs for which developmental toxicity data have not been reported.

## Materials and method

### Materials

BaP (≥ 99%) was obtained from Sigma Aldrich (St.Louis, United States). 3-OHBaP was purchased from TRC Canada (Toronto, Canada). 8-MBaP (≥ 98%) and 3-OH-8-MBaP (≥ 99%) were custom-synthesized at the Biochemical Institute for Environmental Carcinogens (Groβhansdorf, Germany).

The mouse embryonic multipotent stem cell line ES-D3 (CRT-1934™), cell basal medium (SCRR-2011™), and fetal bovine serum (SCRR-30–2020™) were obtained from ATCC (Wesel, Germany). 2 mM L-glutamine and 50 µg/ml penicillin–streptomycin were supplied by Invitrogen (Carlsbad, United States). Cell dissociation solution, 5-fluorouracil (5-FU), 2-mercaptoethanol, non-essential amino acids, cell proliferation reagent WST-1, and murine Leukemia Inhibiting Factor (LIF) were bought from Sigma Aldrich (Schnelldorf, Germany).

Pooled liver S9 fractions from male Sprague Dawley rats were purchased from Tebu-Bio (Heerhugowaard, The Netherlands). Uridine 5’diphosphoglucuronic acid (UDPGA) was obtained from Carbosynth (Compton, United Kingdom). Magnesium chloride, dimethyl sulfoxide (DMSO), trifluoroacetic acid (TFA), and acetonitrile were purchased from Merck (Darmstadt, Germany). Alamethicin and 3’-phosphate 5’-phosphosulfate (PAPS) lithium salt were bought from Sigma Aldrich (Zwijndrecht, The Netherlands).

The reagents used in the animal kinetic study included 5% glucose injection solution (Lot 230308A022) from Qitai animal Health (Qiqihar, China) and polyoxyl castor oil from Shanghai Yuanye Bio-technology Co.,Ltd (Shanghai, China).

### Methods

#### In vitro concentration–response towards 8-MBaP and 3-OH-8-MBaP in the mEST

##### Cell culture

The multipotent mouse ES-D3 cell line was maintained in 0.1% gelatin pre-coated 25 cm^2^ polystyrene cell culture flasks in 5 ml supplemented cell basal medium. The medium was supplemented with 15% fetal bovine serum, 2 mM L-glutamine, 50 µg/ml penicillin–streptomycin, 0.5 mM 2-mercaptoethanol, and 1% non-essential amino acids. The ES-D3 cell line was sub-cultured each 2–3 days and maintained at 37 °C with 5% CO_2_ in a humidified atmosphere. Cell dissociation solution was used to detach the cells. 1000 U/ml LIF was added to keep the cells undifferentiated.

##### ES-D3 cell differentiation and cytotoxicity assay

The developmental toxicity of the test compounds was assessed by the inhibitory effect on the differentiation of cells of the ES-D3 cell line into contracting cardiomyocytes. The assay was performed essentially as previously described (Kamelia et al. 2020). The cells were cultured on the lid of a 96-well plate in 45 hanging drops (volume 20 µL/drop) at a concentration of 37,500 cells/ml. The 96 wells were filled with 250 µL PBS to maintain the humidity. Sterile Eppendorf lids were placed at the corners of the 96-well plate to prevent the hanging drops from contacting the 96-well plate. The plates were sealed with micropore tape and incubated at 37 °C with 5% CO_2_ in a humidified atmosphere for 72 h. After 72 h, 5 ml exposure medium was prepared for each concentration of each test compound with 0.25% DMSO in a 60 ×  15 mm petri dish. The formed embryonic bodies (EBs) in the hanging drops on the lid of the 96-well plate were washed off with exposure medium and collected in the petri dish. The Petri dish was subsequently incubated at 37 °C with 5% CO_2_ in a humidified atmosphere for 48 h. After 48 h, the EBs were transferred to a 24-well plate filled with 1 ml exposure medium per well. Each well contained 1 EB. The 24-well plates were incubated at 37 °C with 5% CO_2_ in a humidified atmosphere for 120 h. The number of EBs with contracting cardiomyocytes and the undifferentiated EBs were counted under a microscope. In the solvent (DMSO) control, the presence of more than 21 EBs with contracting cardiomyocytes out of 24 EBs indicates a valid assay. In each experiment, 0.5 µM (0.065 µg/ml) 5-FU was used as a positive control. The result of the differentiation assay is expressed as the fraction of differentiated EBs of the total number of EBs.

The viability of the ES-D3 cells following 1-day and 5-day exposure to the test compounds was measured with the WST-1 assay. To this end, 100 µL cell suspension was seeded in the inner 60 wells of 96-well plates at a concentration of 2 × 10^5^ cells/ml for 1-day exposure and 10^4^ cells/ml for 5-day exposure and incubated at 37 °C with 5% CO_2_ for 24 h. After 24 h, 100 µL exposure medium with 0.25% DMSO and the required concentration of the test compounds, solvent control, and positive control were added to the cells in triplicate. The plates were incubated at 37 °C with 5% CO_2_ for 1 day or 5 days until measurement. After the exposure period, 20 µL of the cell proliferation reagent WST-1 were added to each well that contained cells and the cells were incubated for 3 h at 37 °C with 5% CO_2_. The absorbance of each plate was measured at 440 nm and 620 nm with a SpectraMax iD3 (San Jose, USA). The result was expressed as cell viability percentage following incubation with the test compounds compared to the solvent control, calculated from the measured absorbance at 440 nm minus the background absorbance at 620 nm.

##### Data analysis

The concentration–response data for the test compounds in the mEST were presented in three curves including curves for 1-day cytotoxicity, 5-day cytotoxicity, and 10-day differentiation assays. The obtained curves were fitted using the inhibition equation log (inhibitor) vs response (three parameters) option using GraphPad Prism software 5.0 (California, USA). The concentration of the test compound that resulted in 50% inhibitory effect on differentiation and cytotoxicity was derived and expressed as IC_50_.

#### Obtaining kinetic parameters for metabolism of 8-MBaP and 3-OH-8-MBaP

The oxidative metabolism of 8-MBaP mediated by P450 enzymes in rat liver microsomes was characterized in our previous study (Wang et al. 2022). The kinetic parameters including K_m_ and V_max_, obtained by fitting the Michaelis–Menten equation, for formation of 3-OH-8-MBaP and for formation of the sum of all other metabolites were used in the PBK model developed for 8-MBaP and are presented in Table 1.

**Table 1:** Kinetic parameter values for liver metabolism of 8-MBaP and 3-OH-8-MBaP in rat

Reaction	Kinetic parameters			
	V_max_	K_m_^*b*^	Scaled V_max_^*c*^	Scaled V_max_^d^
8-MBaP to 3-OH-8-MBaP*	0.07^a^	11.55	0.19	2
8-MBaP to other metabolites*	0.43^a^	8.07	1.18	11
Glucuronidation of 3-OH-8-MBaP	6.92^e^	15.58	51.9	470
Sulfation of 3-OH-8-MBaP	0.15^e^	26.07	1.16	10

^*^Data were derived from the study ofWang et al (2022)

^a^nmol/min/mg microsomal protein

^b^µM

^c^µmol/h/g liver

^d^µmol/h/liver

^e^nmol/min/mg S9 protein

##### Glucuronidation of 3-OH-8-MBaP

The conjugation of 3-OH-8-MBaP with glucuronic acid was studied in incubations with rat liver S9 in vitro. The incubation time and S9 protein concentration were optimized to ascertain linearity of glucuronidation with time and the amount of S9 protein. The incubation was performed with a 200 µL mixture in glass vials, and consisted of (final concentrations) 0.1 mM Tris buffer (pH 7.4), 0.1 mg/ml pooled rat liver S9, 3 mM UDPGA, 5 mM MgCl_2_, 0.025 mg/ml alamethicin, and 0–100 µM 3-OH-8-MBaP. No metabolites were formed in control incubations without either rat liver S9 or co-factor UDPGA. The final concentration of substrate vehicle (DMSO) in the incubation mixture was 1% (v/v). The incubation mixtures without substrate were pre-incubated at 37 °C for 1 min. The reaction was initiated by adding substrate to the incubation mixtures that were subsequently incubated at 37 °C for 20 min, after which the reaction was terminated by adding 100 µL ice-cold acetonitrile. The incubation mixture was subsequently centrifuged at 3717*g* at 4 °C for 5 min. The supernatant was collected for ultra-performance liquid chromatography (UPLC) analysis.

##### Sulfation of 3-OH-8-MBaP

The conjugation of 3-OH-8-MBaP by sulfation was studied in incubations with rat liver S9 in vitro. The incubation time and S9 protein concentration were optimized to ascertain linearity of the sulfation reaction with respect to time and amount of protein. The 200 µL incubation mixture consisted of (final concentrations) 0.1 mM Tris buffer (pH 7.4), 0.1 mg/ml rat liver S9, 0.2 mM PAPS and 0–100 µM 3-OH-8-MBaP. No metabolites were detected in control incubations without either rat liver S9 or co-factor PAPS. After 1-min pre-incubation of the incubation mixture without substrate, the reaction was initiated by adding substrate from a 100 times concentrated stock solution in DMSO (final concentration 1% DMSO (v/v)). The incubation mixture was incubated at 37 °C for 70 min after which 100 µL ice-cold acetonitrile was added to the incubation mixture to terminate the reaction. The incubation mixture was subsequently centrifuged at 3717*g* at 4 °C for 5 min. The supernatant was collected for subsequent UPLC analysis.

##### UPLC analysis

An Acquity UPLC system with a photodiode array (PDA) detector was used to quantify the metabolites formed in the glucuronidation and sulfation incubations. The supernatants of the incubation samples were injected onto an Acquity UPLC BEH® C18 column (21 × 50 mm, 1.7 µm, Waters, Milford, MA). The total running time per sample was 22 min with a flow rate of 0.6 ml/min. The temperature of column and auto sampler was 40 °C and 10 °C, respectively. The injection volume was 3.5 µL and the detection wavelength ranged from 190 to 400 nm. Two eluents were used including A (nanopure with 0.1% (v/v) TFA) and B (acetonitrile with 0.1% (v/v) TFA). The gradient elution started from 90% A and 10% B applied from 0.0 min to 0.5 min, which was changed to 10% A and 90% B from 0.5 to 15.5 min and then kept at 10% A and 90% B from 15.5 min to 18.5 min, and changed back to 90% A and 10% B from 18.5 to 22.0 min. Metabolites were quantified as previously described in our study on microsomal metabolism of BaP and several of its methyl substituted analogs (Wang et al 2022).

##### Data analysis

The concentration of the glucuronidated and sulfated metabolites was further used to calculate the velocity of the enzymatic reaction expressed in nmol/min/mg S9 protein. The curve reflecting the substrate (3-OH-8-MBaP) concentration-dependent velocity of the metabolite formation was fitted by the Michaelis–Menten equation using GraphPad Prism 5 (California, USA). The kinetic constants derived included the K_m_ (µM) and V_max_ (nmol/min/mg S9 protein).

#### PBK model for 8-MBaP in rats

**Fig. 2:**
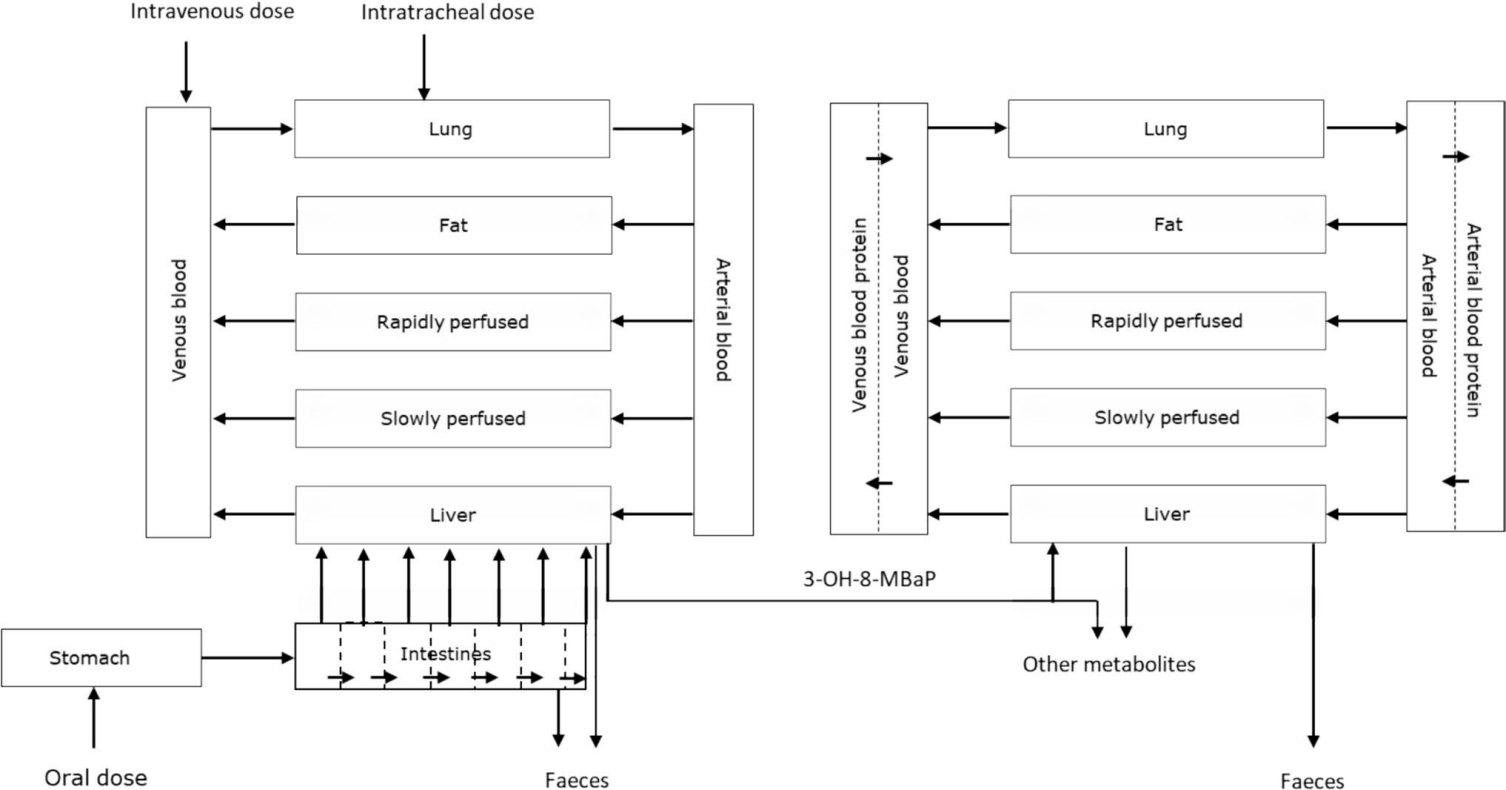
Structure of the PBK model of 8-MBaP with a sub-model for 3-OH-8-MBaP in rats, in analogy to the PBK model previously developed and validated for BaP and its active metabolite 3-OHBaP (Wang et al. 2021)

The PBK model for 8-MBaP was developed based on the model for BaP that was previously validated by comparison of predictions made to available in vivo toxicokinetic data (Wang et al. 2021). Figure 2 presents the conceptual model that simulates absorption, distribution, metabolism, and excretion of 8-MBaP in rats. The model includes several organ and tissue compartments that are connected to the systematic circulation. The model of 8-MBaP contains a sub-model for 3-OH-8-MBaP since the developmental toxicity of 8-MBaP needs bioactivation to 3-OH-8MBaP as shown in the present study by results obtained in the mEST.

Exposure to 8-MBaP was described in the PBK model for the intravenous, intratracheal, and oral route. The uptake of 8-MBaP was assumed to be complete and to occur with (1) a transport rate constant Kd = 1000000h^−1^ from needle to blood, (2) an absorption rate constant Kt = 1h^−1^ from trachea to lung, and (3) an absorption rate constant Ka = 1h^−1^ from stomach to gastrointestinal (GI) tract followed by uptake from the GI tract compartments into the liver. The absorption rate constant kabin of 0.003 L/h was calculated as P_app_*SA_in_ with P_app_ being the apparent intestinal permeability coefficient in vivo and SA_in_ being 0.134 dm^2^ per intestine compartment (Sun et al. 2002; Zhang et al. 2018). The intestine was divided into seven sub-compartments (Wang et al. 2021; Zhang et al. 2018). The permeability coefficient (P_app_) of 8-MBaP was assumed to be the same as the one for BaP being 0.02 dm/hr (Sun et al. 2002).

In liver, 8-MBaP was assumed to be metabolized by cytochrome P450 enzymes to 3-OH-8-MBaP and other metabolites, including the side-chain hydroxylated metabolite 8-hydroxymethyl-BaP (8-OH-8MBaP) and dihydrodiols (Fig. 1) (Wang et al. 2022). 3-OH-8-MBaP was subsequently modeled to be conjugated by glucuronidation and sulfation in the liver. The kinetic constants for these conjugation reactions were determined in incubation experiments described in Sect. “Obtaining kinetic parameters for metabolism of 8-MBaP and 3-OH-8-MBaP”, the kinetic constants for the microsomal cytochrome P450 catalyzed reactions were taken from literature (Wang et al. 2021). The scaling factors from rat liver microsomes and rat liver S9 to the whole liver were 45 mg microsomal protein/g liver and 125 mg S9 protein/g liver, respectively (Houston and Galetin 2008). The oxidative metabolism of 8-MBaP and conjugation of 3-OH-8-MBaP in lung was considered to be negligible, based on what was found for BaP (Wang et al. 2021).

Table 2 summarizes the physiochemical parameters of 8-MBaP and 3-OH-8-MBaP and the physiological parameter values including body weight, fractional tissue volumes, and fractional tissue blood flow of rats (Brown et al. 1997; Crowell et al. 2011; Marie et al. 2010; Moreau and Bouchard 2015) used in the PBK model. The tissue:blood partition coefficients of 8-MBaP and 3-OH-8-MBaP were calculated using the Berezhkovskly method in the quantitative in vitro to in vivo extrapolation (QIVIVE) tool developed by Wageningen Food Safety Research (WFSR) (https://wfsr.shinyapps.io/wfsrqivivetools/) (Berezhkovskiy 2004; Punt et al. 2021), and are also presented in Table 2.

**Table 2:** Physiological, anatomical, and physicochemical parameter values for 8-MBaP and 3-OH-8-MBaP for the rat PBK model

Model parameter	Symbol	Value	Reference
Physiological parameters			
Body weight Fractional tissue volumes	BW	0.245^a^	Marie et al. (2010) Moreau and Bouchard (2015)
Fat	VFc	0.065	DeJongh et al. (1997)
Liver	VLc	0.037	
Lung	VLuc	0.005	
Arterial blood	VABc	0.0257	
Venous blood	VVBc	0.0514	
Rapidly perfused tissue	VRc	0.2159	
Slowly perfused tissue	VSc	0.6	
Cardiac output (mL/s)	QC	15*BW^0.74^	
Fractional tissue blood flows			
Fat	QFc	0.07	
Liver	QLc	0.183	
Lung	QLuc	1	
Rapidly perfused tissue	QRc	0.4	
Slowly perfused tissue	QSc	0.347	
Physicochemical parameters			
*8-Methyl-benzo[a]pyrene*			
Molecular weight	MW8MBaP	266.34	
LogP		6.7	Cheng et al. (2007)
Fraction unbound	*f*_ub_	0.003	Calculated according to Lobell and Sivarajah (2003)
Tissue:blood partition coefficients			
Fat	PF8MBAP	434.55	Berezhkovskiy (2004)
Liver	PL8MBAP	12.85	
Lung	PLu8MBAP	14.65	
Rapidly perfused tissue	PR8MBAP	12.85	
Slowly perfused tissue	PS8MBAP	7.13	
*3-Hydroxy-8-methyl-benzo[a]pyrene*			
Molecular weight	MW3OH8MBaP	282.34	
LogP		6.3	Cheng et al. (2007)
Fraction unbound	*f*_ub_	0.004	Calculated according to Lobell and Sivarajah (2003)
Tissue:blood partition coefficients			
Fat	PF3OH8MBAP	392.83	Berezhkovskiy (2004)
Liver	PL3OH8MBAP	12.55	
Lung	PLu3OH8MBAP	14.31	
Rapidly perfused tissue	PR3OH8MBAP	12.55	
Slowly perfused tissue	PS3OH8MBAP	6.98	

^a^Median of body weights of rats in these two studies

Given the lipophilic properties of 3-OH-8-MBaP, a blood protein compartment was included to prevent 3-OH-8-MBaP from partitioning into the fat tissue. The unbound fraction (fub, in vivo) of 3-OH-8-MBaP in the blood compartment was calculated using the Lobell and Sivarajah method in the previously mentioned QIVIVE tool developed by WFSR (Lobell and Sivarajah 2003). Biliary excretion of 8-MBaP and 3-OH-8-MBaP to feces was included with an assumed excretion rate constant Kb = 1 h^−1^ from liver to feces.

#### Evaluation of the PBK model for 8-MBaP

The predictions from the developed PBK model for 8-MBaP were evaluated in two ways. First the model was validated based on read-across from BaP for which in vivo kinetic data on blood concentrations of the parent PAH and its 3-OH metabolite were available. These data were previously used to validate the model for BaP showing the conceptual PBK model to be adequate (Wang et al. 2022) and providing a basis for its use in the present study for 8-MBaP. In the present study, the predictions for 8-MBaP and its metabolite 3-OH-8-MBaP were compared to the same set of available in vivo kinetic data (Marie et al. 2010; Moreau and Bouchard 2015). In addition, the PBK models were validated by comparison of the predictions made to results from an additionally performed in vivo kinetic study that quantified BaP and 8-MBaP blood concentrations upon an intravenous dose of 50 mg/kg bw BaP or 8-MBaP, performed as described below.

##### In vivo* kinetic study: animals, treatment, and sampling*

Six male Sprague–Dawley rats at the age of 8–9 weeks weighing 286-305g/rat were obtained from Charles River Inc (Beijing, China). The rats were maintained in plastic cages in two groups of three in a temperature-controlled (ranging from 20 °C to 25 °C) and humidity-controlled (ranging from 40–70%) environment on a 12-h light–dark cycle. The animal bedding was corncob and used after autoclaving. The bedding and the cages were changed twice a week. The number of animals was restricted to 3 per dose group and control blood samples were collected before dosing. Feeding and living conditions followed the Chinese Laboratory Animal-Requirement of environment and housing facilities (GB14925-2010). The present rat study was approved by Institutional Animal Care and Use Committee (IACUC-BF202305006).

All animals were fasted 8 h before intravenous injection and resumed feed supply 4 h after dosing. One group of rats was exposed intravenously to 50 mg/kg bw BaP and the other group of rats was exposed to 50 mg/kg bw 8-MBaP to compare the predicted toxicokinetics by the developed PBK models. The injection vehicle of BaP and 8-MBaP was 20% polyoxyl castor oil: 80% glucose solution. Rats received a single dose of 50 mg/kg bw by administering 3 ml 16.67 mg/ml of either BaP or 8-MBaP.

After administration, blood samples (0.1–0.2 ml blood volume) were taken from the medial canthus venous plexus of the animal and anticoagulated with sodium heparin. Prior to orbital venipuncture and blood collection, the animals were anesthetized by CO_2_ inhalation (70% CO_2_ + 30% O_2_). Blood was sampled at 1 min, 0.5, 1, 2, 4, 8, 12, 24, 32, 48, and 72 h post-dosing. After collection, blood samples were frozen and were stored at −20 °C until analysis. All animals were euthanized with 100% CO_2_ after the experiment.

##### Extraction and bioanalysis of the blood samples

Aliquots of 100 µL of blood samples were transferred into centrifuge tubes and 700 µL sodium acetate buffer (0.1M, pH 5.0) and 6 µL β-glucuronidase-arylsulfatase were added. The samples were incubated for 1 h at 37 °C and subsequently extracted twice with 800 µL ethyl acetate saturated with water. The samples were shaken for 30 min and centrifuged 5 min at 2000 g (4 °C). The upper organic phase was transferred to a centrifuge tube and evaporated under a gentle stream of nitrogen at 40 ℃ in a water bath. The residues were re-dissolved with 200 µL ascorbic acid:methanol (1:100).

In the re-dissolved samples, BaP and 8-MBaP and their metabolites were quantified by liquid chromatography using an Agilent 1200 chromatograph coupled with a fluorescence detector (Santa Clara, USA). The quantification of BaP and 8-MBaP and their metabolites was based on the respective calibration curve of the reference chemical in blood extracted by the same procedure. The analytes were separated using a ZORBAX Eclipse xdb-c180 column (4.6 mm × 300 mm × 5 µm, Agilent, Santa Clara, USA) with a total run time of 15 min at a flow rate of 1 ml/min. The injection volume was 20 µL and the column temperature was maintained at 25°C. Eluent A was acetonitrile and eluent B was 0.1% formic acid in water. The detection excitation and emission wavelengths were set at 382 nm and 441 nm, respectively. The gradient elution started from 75% A and 25% B applied from 0.0 min to 5.5 min, which was changed to 95% A and 5% B from 5.5 to 6.0 min, and 95% A and 5% B was kept from 6.0 min to 13.0 min and changed to 75% A and 25% B from 13.0 to 13.5 min, and finally changed to 25% A and 75% B from 13.5 to 15 min.

##### Data analysis

The relationship between blood concentration of 8-MBaP and the formed metabolite 3-OH-8-MBaP in rats was obtained. For comparison, the same relationship was derived for BaP and 3-OHBaP. The curves describing the time-dependent blood concentration of BaP and 8-MBaP and their metabolites were fitted with GraphPad Prism software 5.0 (California, USA).

#### Sensitivity analysis of the PBK model for 8-MBaP

A sensitivity analysis was performed to determine the influential parameters for the PBK model predicted maximum blood concentration (C_max_) of 3-OH-8-MBaP. Three exposure routes including intravenous, intratracheal, and oral uptake of 8-MBaP were evaluated at the dose of 10 mg/kg bw. The normalized sensitivity coefficients (SC) were calculated using the following Eq. (1):

1 $$SC\, = \,\left\{ {\left( {C^{\prime}{-}C} \right)/\left( {P^{\prime}{-}P} \right)} \right\} \cdot \left( {P/C} \right)$$

In this formula, P represents the initial value of a parameter and P’ the modified value with 10% increase. C represents the initial value of C_max_ of 3-OH-8-MBaP and C’ the modified value caused by the 10% increase of P. Each parameter was modified independently while other parameters remained unchanged. When changing the fractional tissue blood flows, the total blood flow of rat, liver, rapidly perfused tissue and slowly perfused tissue was maintained at 1.

#### Reverse dosimetry and read-across to predict developmental toxicity of 8-MBaP

The in vivo dose–response curve for the developmental toxicity of 8-MBaP was assumed to be dependent on the C_max_ of unbound 3-OH-8-MBaP in the maternal rat blood (C_ub, in vivo_). It was reported that the blood concentration of BaP in maternal and fetal blood are comparable in pregnant rats (Withey et al. 1993); therefore, the same assumption was applied for 8-MBaP eliminating the need to include a placental barrier and separate fetal compartment in the model. The relationship between the in vivo 3-OH-8-MBaP concentration in maternal blood (C _in vivo_) and the in vitro 3-OH-8-MBaP concentration in the mEST (C_in vitro_) was described by the following Eq. (2):

2 $$C_{in \, vivo} *f_{ub, \, in \, vivo} \, = \,C_{in \, vitro} * \, f_{ub, \, in \, vitro}$$

The fraction unbound in blood (*f*_ub, in vivo_) of 3-OH-8-MBaP was calculated as 0.004 (Table 2) based on the methodology mentioned in Sect. “PBK model for 8-MBaP in rats”. The fraction unbound in the mEST (*f*_ub, in vitro_) was assumed to be half of the *f*_ub, in vivo_ based on the ratio between the protein content in rat blood plasma (7.5%) and the mEST assay medium (15%). The unbound concentration in vitro (C_ub, in vitro_) inducing the developmental toxicity response in mEST was converted using Eq. (2) to an unbound concentration in blood (C_ub,in vivo_) that was further converted by the PBK model to a predicted oral dose.

## Results

### In vitro concentration–response of 8-MBaP and 3-OH-8-MBaP in the mEST

**Fig. 3:**
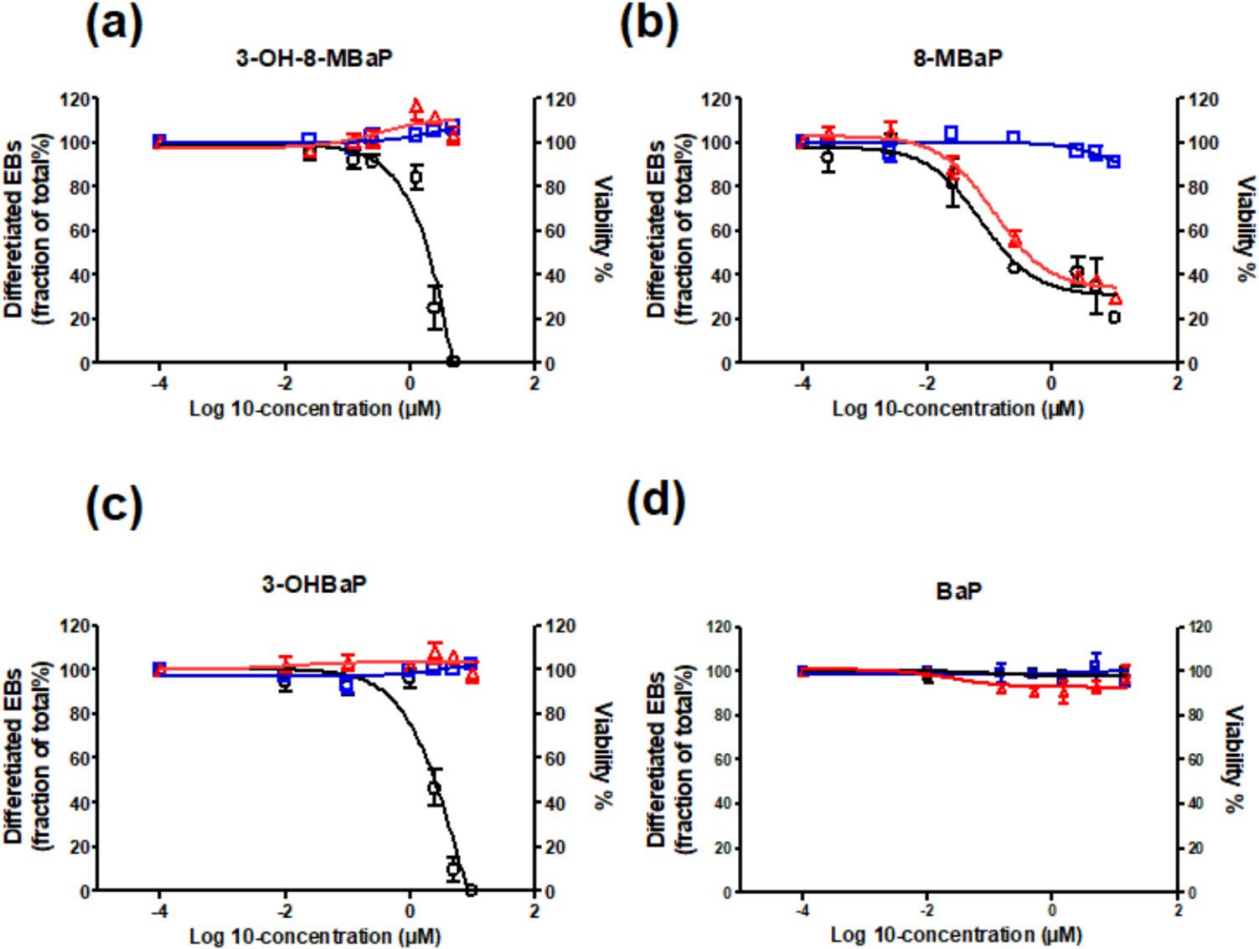
Concentration–response curves for the effect of **a** 3-OH-8-MBaP, **b** 8-MBaP, **c** 3-OHBaP, and **d** BaP on differentiation of ES-D3 cells into beating cardiomyocytes (black open circles and black solid lines), and the accompanying 1-day cytotoxicity (blue open squares and blue solid lines), and 5-day cytotoxicity (red open triangles and red solid lines). The presented data are the result from at least three independent experiments, shown as mean ± standard error of the mean (SEM) (color figure online)

The developmental toxicity of 8-MBaP and 3-OH-8-MBaP was assessed in vitro by quantifying the inhibitory effect of these model compounds on the differentiation of ES-D3 cells into beating cardiomyocytes in the mEST. The concentration–response curves for the cytotoxicity and differentiation assays of the mEST for (a) 3-OH-8-MBaP and (b) 8-MBaP as well as for (c) 3-OHBaP and (d) BaP are presented in Fig. 3. 8-MBaP appeared to cause a reduction in both differentiation and cell viability on Day 5 with an IC_50_ of 0.83 µM and 1.74 µM, respectively, indicating the effect on differentiation to be mainly due to cytotoxicity instead of reflecting developmental toxicity of 8-MBaP. For 3-OH-8-MBaP, a concentration-dependent inhibition of the number of beating cardiomyocytes was observed with an IC_50_ of 1.40 µM without accompanying cytotoxicity. 3-OHBaP inhibits ES-D3 cell differentiation with an IC_50_ of 1.51 µM in a concentration-dependent manner at non-cytotoxic concentrations, while BaP itself is inactive in this differentiation assay.

### Kinetics of 8-MBaP and 3-OH-8-MBaP in rats

The kinetics for the cytochrome P450-mediated metabolism of 8-MBaP in rats was described for the formation of 3-OH-8-MBaP and for the sum of other metabolites. The kinetic parameters for formation of 3-OH-8-MBaP and for formation of the sum of other metabolites are presented in Table 1, and were obtained from our previous study (Wang et al. 2022). The V_max_ values for 3-OH-8-MBaP formation and for the sum of the formation of other metabolites were 0.07 and 0.43 nmol/min/mg microsomal protein, respectively, amounting to 2 and 11 µmol/h/liver using the scaling factor described in Sect. “PBK model for 8-MBaP in rats” and converting minutes to hours, nmol to μmol and assuming a liver weight of 9.1 g.

The UPLC analysis of the microsomal or S9 incubations showed an elution time for 3-OH-8-MBaP of 9.32 min with a maximum wavelength of 260.8 nm. In the incubations with 3-OH-8-MBaP as substrate, pooled rat liver S9 and UDPGA as co-factor, the glucuronide metabolite was detected at a retention time of 6.56 min with a maximum wavelength of 306.2 nm representing a peak that was not present in the control without UDPGA. In the incubations with 3-OH-8-MBaP as substrate, pooled rat liver S9 and PAPS as co-factor, the sulfated metabolite eluted at 6.60 min with a maximum wavelength at 304.3 nm.

**Fig. 4:**
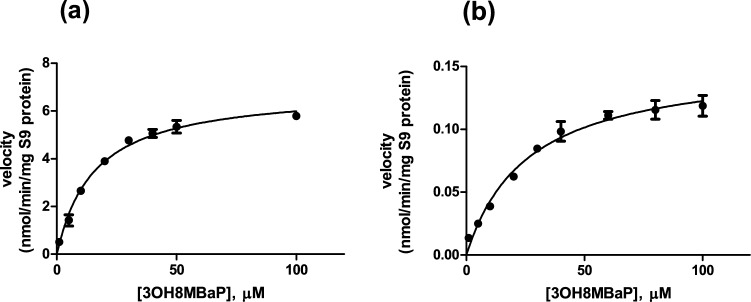
Substrate concentration-dependent kinetic curves of **a** glucuronidation and **b** sulfation of 3-OH-8-MBaP in incubations with rat liver S9 and the respective cofactors. Symbols represent the mean of three independent experiments, and the error bars represent the SEM

The substrate concentration-dependent glucuronidation and sulfation of 3-OH-8-MBaP in incubations with rat liver S9 are shown in Fig. 4. The kinetic curves were fitted with the Michaelis–Menten equation to define the parameters V_max_ and K_m_ that are presented in Table 1. The V_max_ of the hepatic glucuronidation and sulfation of 3-OH-8-MBaP were 6.92 and 0.15 nmol/min/mg S9 protein, respectively, amounting to 470 and 10 µmol/h/liver using the scaling factor described in Sect. “PBK model for 8-MBaP in rats” and converting minutes to hours, nmol to µmol and assuming a liver weight of 9.1 g.

### Evaluation of the PBK model of 8-MBaP

**Fig. 5:**
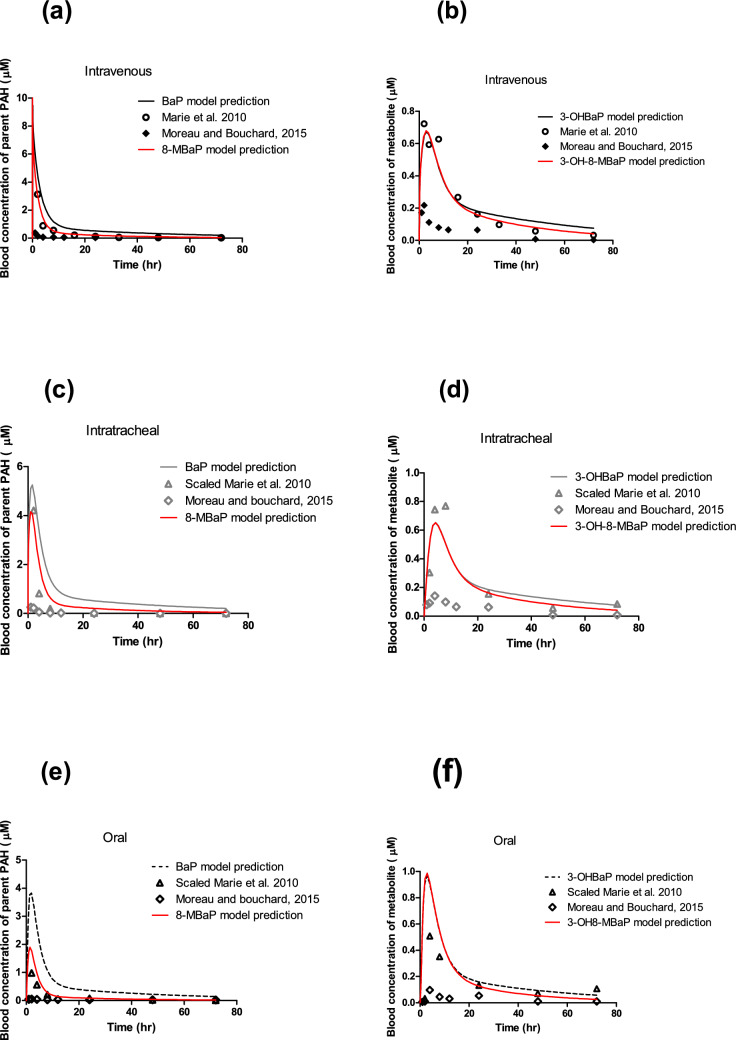
PBK model-based predicted time-dependent blood concentration of 8-MBaP and 3-OH-8-MBaP in rats (red curves) and, for comparison, the previously PBK model-predicted blood concentrations for the structurally related substance BaP and its metabolite 3-OHBaP. The dose level was 10 mg/kg bw in all studies. Figure **a** and **b** show the predicted blood concentrations of 8-MBaP and 3-OH-8-MBaP (red solid lines), respectively, and that of BaP and 3-OHBaP (black solid lines), together with the reported in vivo data of BaP and 3-OHBaP from Marie et al. (black open circles) (2010) and Moreau and Bouchard (black filled diamond) (2015) upon intravenous exposure. Figure **c** and **d** present the predicted blood concentrations of 8-MBaP and 3-OH-8-MBaP (red solid lines), respectively, and, for comparison, those of BaP and 3-OHBaP (grey solid lines), together with the reported in vivo data of BaP and 3-OHBaP from Marie et al. (grey open triangles) (2010) and Moreau and Bouchard (grey open diamond) (2015) upon intratracheal exposure. Figure **e** and **f** demonstrate the predicted blood concentrations of 8-MBaP and 3-OH8-MBaP (red solid lines), respectively, and that of BaP and 3-OHBaP (black dashed lines), together with the reported in vivo data of BaP and 3-OHBaP from Marie et al. (black open triangles) (2010) and Moreau and Bouchard (black open diamond) (2015) upon oral exposure. The results obtained reveal that the prediction for 8-MBaP and 3-OH-8-MBaP, in spite of differences in the kinetics for metabolism and log Kow values that dominate distribution, are close to the predictions for BaP and its metabolite 3-OHBaP and also in line with the in vivo data available for BaP and 3-OHBaP (color figure online)

The newly developed PBK model of 8-MBaP in rats was first evaluated by comparison of the reported in vivo and the predicted time-dependent blood concentration of BaP and 3-OH-BaP with the predictions for the blood concentration of 8-MBaP and 3-OH-8-MBaP. Figure 5 shows the blood concentration of BaP and 3-OH-BaP upon (a) intravenous, (b) intratracheal, (c) oral exposure to 10 mg BaP/kg bw in rats in comparison with the predictions for blood concentration of 8-MBaP and 3-OH-8-MBaP upon an equimolar dose of 8-MBaP. The predicted time-dependent blood concentration curves of 8-MBaP and 3-OH-8-MBaP were well in line with the predictions and the data reported in the literature for BaP and 3-OHBaP. C_max_ represents the peak concentration of the test substance in the blood, which was used for the validation. During developmental stages, embryos and fetuses may be particularly sensitive to peak concentration of the test substance. For chemicals that cause developmental toxicity, the peak concentration is more relevant than overall exposure (AUC) as the C_max_ can trigger immediate adverse effects. Table 3 presents the maximum blood concentration C_max_ of 8-MBaP and 3-OH-8-MBaP in comparison to BaP and 3-OHBaP predicted by the PBK models. The C_max_ of 8-MBaP was predicted to be comparable and only somewhat lower than that obtained upon an equimolar dose of BaP, being 0.95-, 0.77- and 0.48-fold times the C_max_ of BaP upon intravenous, intratracheal, and oral exposure, respectively. The C_max_ predicted for 3-OH-8-MBaP for each exposure route was identical to that predicted for 3-OHBaP resulting in a ratio of 1.0. The somewhat lower Cmax values for 8-MBaP than for BaP accompanied by comparable C_max_ values for their 3-OH metabolites can be ascribed to faster overall metabolic clearance of 8-MBaP due to the contribution of methyl side chain oxidation, while the rate of aromatic hydroxylation at C3 was only somewhat less effective for 8-MBaP than for BaP.

**Table 3:** Maximum blood concentration (C_max_) of 3-OH-8-MBaP in rats predicted by the PBK model for 8-MBaP in comparison to C_max_ of 3-OHBaP predicted by the model for BaP upon intravenous, intratracheal, and oral exposure of rats to 10 mg/kg bw

Route of exposure	C_max_ 3-OH-8-MBaP, µM	C_max_ 3-OHBaP, µM	C_max_ 3-OH-8-MBaP/C_max_ 3-OHBaP	C_max_ 8-MBaP, µM	C_max_ BaP, µM	C_max_ 8-MBaP/C_max_ BaP
Intravenous	0.68	0.68	1.0	728.1	768.5	0.95
Intratracheal	0.65	0.65	1.0	3.97	5.14	0.77
Oral	0.97	0.96	1.0	1.79	3.75	0.48

**Fig. 6:**
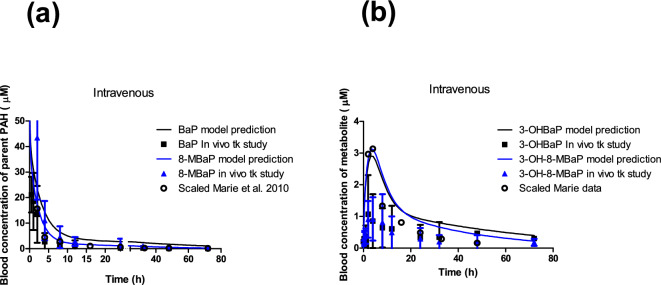
PBK model-based prediction for time-dependent blood concentrations of a) 8-MBaP (blue curves) and BaP (black curves) and of b) 3-OH-8-MBaP (blue curves) and 3-OH-BaP (black curves) upon intravenous exposure of rats to 50 mg/kg bw 8-MBaP or BaP. For comparison, the measured in vivo blood concentrations in time of a) 8-MBaP (blue triangles) and BaP (black squares) and of b) 3-OH-8-MBaP (blue triangles) and 3-OHBaP (black squares) are presented. The error bars represent the standard deviation (SD) of the mean. The intravenous data from Marie et al. (2020) were scaled to 50 mg/kg bw assuming linear dose dependency, and are shown as open circles for a) BaP and b) 3-OHBaP. The zoom-in graphs for x axis from 0–5 h are presented in Fig. 1 Supplementary Material 2 (color figure online)

To further validate the PBK model, an in vivo kinetic study was performed in which rats were dosed with 50 mg/kg bw 8-MBaP or BaP via intravenous exposure. Time-dependent blood concentrations of 8-MBaP and BaP and those of their active metabolites 3-OH-8-MBaP and 3-OHBaP, respectively, were quantified over a period of 72 h. The results obtained are presented in Fig. 6a and b. Taking into account the fivefold higher dose level (10 vs 50 mg/kg bw), the results obtained match those of Marie et al. (2010) (Fig. 6). Figure 6 also presents the PBK model-predicted blood concentration versus time profiles. For this comparison, it follows the Marie et al. data (Fig. 5a and b) where it was observed that the PBK model predicts the blood concentration of BaP quite well, while it somewhat overpredicts the 3-OHBaP values with the blood concentration of BaP and the predicted concentrations were 1.6- to 6.7-fold (*t* = 1–12 h) higher than what was observed in the animal study. The model sufficiently predicted the blood concentration of 8-MBaP which was 2.9 µM and 3.1 µM for 3-OHBaP and 3-OH-8-MBaP, respectively. The observed in vivo Cmax of 3-OHBaP and 3-OH-8-MBaP was 2.6-fold and 3.4-fold lower than the model prediction. The results obtained reveal that the prediction for 8-MBaP and 3-OH-8-MBaP, in spite of differences in the kinetics for metabolism and log Kow values that dominate distribution, is close to the predictions for BaP and its metabolite 3-OHBaP and also in line with the in vivo data available for BaP and 3-OHBaP.

### Sensitivity analysis of the PBK model of 8-MBaP

**Fig. 7:**
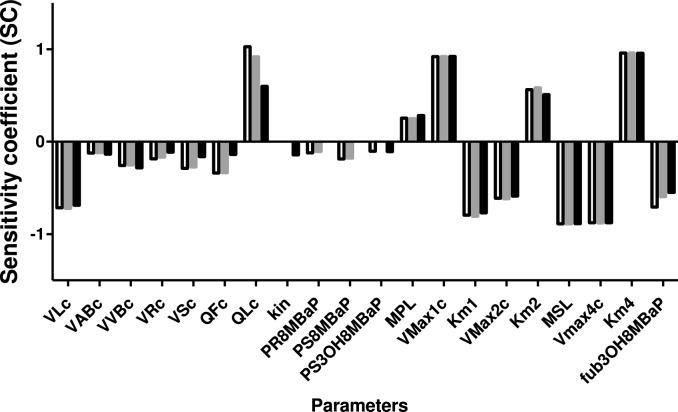
Sensitivity coefficients (SC) of PBK model parameters for the predicted C_max_ of 3-OH-8-MBaP in rat blood after intravenous (white open bars), intratracheal (grey filled bars), or oral (black filled bars) administration of 10 mg/kg bw 8-MBaP. Model parameters with an absolute SC of ≥ 0.1 are shown. VLc = fraction of liver tissue, VABc = fraction of arterial blood, VVBc = fraction of venous blood, VRc = fraction of rapidly perfused tissue, VSc = fraction of slowly perfused tissue, QFc = fraction of blood flow to fat, QLc = fraction of blood flow to liver, kin = transfer rate to next compartment within the intestines, PR8MBaP = rapidly perfused tissue:blood partition coefficient of 8-MBaP, PS8MBaP = slowly perfused tissue: blood partition coefficient of 8-MBaP, PS3OH8MBaP = slowly perfused tissue:blood partition coefficient of 3-OH-8-MBaP, MPL = microsomal protein content in liver, Vmax1c = maximum rate of 3-OH-8-MBaP formation in liver, Km1 = Michaelis–Menten constant for metabolism of 8-MBaP to 3-OH-8-MBaP in liver, Vmax2c = maximum rate formation of other metabolites in liver, Km2 = Michaelis–Menten constant for metabolism of 8-MBaP to other metabolites, MSL = S9 protein content in liver, Vmax4c = maximum rate of glucuronidation of 3-OH-8-MBaP formation in liver, Km4 = Michaelis–Menten constant for glucuronidation 3-OH-8-MBaP in liver, fub3OH8MBaP = fraction unbound of 3-OH-8-MBaP (color figure online)

Figure 7 demonstrates the results of a sensitivity analysis to define the parameters that influence the predictions of in vivo blood concentration of 3-OH-8-MBaP at a dose of 10 mg/kg bw 8-MBaP to the largest extent. The calculated sensitivity coefficients (SCs) are presented for intravenous (white open bars), intratracheal (grey filled bars), and oral (black filled bars) exposure. Only the parameters with normalized absolute SCs that were greater than 0.1 are presented. The most influential parameters with an absolute SC value higher than 0.5 include the fractional tissue volume of liver (VLc), the fractional tissue blood flow to liver (QLc), the metabolic parameters for oxidative conversion of 8-MBaP to 3-OH-8-MBaP and of 8-MBaP to other metabolites (V_max1c_, Km_1_, V_max2c_, K_m2_), the S9 protein content of the liver (MSL), the metabolic parameters for glucuronidation of 3-OH-8-MBaP (V_max4c_, K_m4_), and the value of the fraction unbound of 3-OH-8-MBaP (f_ub, in vivo_).

### Prediction of in vivo dose–response curves from in vitro concentration–response curves

**Fig. 8:**
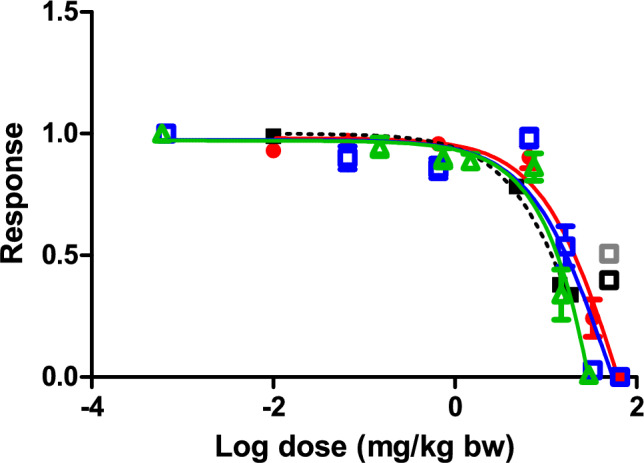
The predicted dose–response curves for developmental toxicity of 8-MBaP (green open triangles and green solid line) and BaP (blue open squares and blue solid line) obtained by reverse dosimetry of the mEST differentiation data reported in the present study for 3-OH-8-MBaP (Fig. 3a) and 3-OH-BaP (Fig. 3c), in comparison to the predicted dose–response curve previously predicted by the same method for BaP (red filled circle and red solid line) and the dose–response data reported by Archibong et al. (2002) for an in vivo developmental toxicity study for BaP (black filled squares and black dashed line). The data from the in vivo study reported by Bui et al. (1986) are shown as a grey open square for 6-day exposure and as a black open square for 3-day exposure. Each symbol represents the mean of the response and the error bars represent the standard error of the mean (SEM) (color figure online)

Based on the developed PBK model of 8-MBaP following the oral route, the in vitro concentration–response curve for the effect of 3-OH-8-MBaP on cell differentiation in the mEST (black curve in Fig. 3a) was translated to an in vivo dose–response prediction for the developmental toxicity of 8-MBaP (Fig. 7). The predicted oral doses of 8-MBaP were calculated from the in vitro concentration of 3-OH-8-MBaP in the mEST using the equations provided in Sect. “Reverse dosimetry and read-across to predict developmental toxicity of 8-MBaP” with correction for the difference in protein binding in the in vitro and in vivo situation. The half maximal effect dose ED_50_ value derived from of the predicted dose–response curve for 8-MBaP is presented in Table 4 together with the previously reported predicted and experimental ED_50_ values for BaP. The predicted ED_50_ value for BaP of 14.0 mg/kg bw (red line in Fig. 8) (Wang et al. 2021) was 1.3-fold higher than the ED_50_ value for 8-MBaP that was derived from the predicted dose–response curve for single oral exposure to 8-MBaP, being 10.5 mg/kg bw (green curve in Fig. 8). In addition, the concentration–response curve obtained in the present study for 3-OHBaP in the mEST (black curve in Fig. 3c) was also translated to a dose–response prediction for BaP using the published PBK model of BaP (Wang et al. 2021). The ED_50_ of BaP, thus, obtained from the predicted in vivo dose–response curve (blue curve in Fig. 8) was 14.0 mg/kg bw and only 0.9-fold lower than the ED_50_ of 15.1 mg/kg bw derived from the previously reported predicted dose–response curve for BaP (red curve in Fig. 8) (Wang et al. 2021) and 1.3-fold higher than the value derived from the reported in vivo study on developmental toxicity of BaP of 10.8 mg/kg bw (black dotted line in Fig. 8) (Archibong et al. 2002).

**Table 4:** ED_50_ obtained from the predicted dose–response curve of 8-MBaP in comparison to the ED_50_ from the model prediction and published in vivo data for BaP upon oral exposure in rats

Study	The present study model prediction		Wang et al. 2021 model prediction	Archibong et al. 2002 In vivo toxicity study
Compound	8-MBaP	BaP	BaP	BaP
ED_50_ (mg/kg bw)	10.5	14.0	15.1	10.8

The dose–response curves were constrained with bottom (constant equal to 0) and top (constant equal to 100), when deriving the ED_50_ values

## Discussion

The present study developed a PBK model for 8-MBaP in rats, a methylated analog of BaP, using read-across from the previously developed and well-validated model for non-substituted BaP. The current model includes intravenous, intratracheal, and oral exposure routes. Both models of 8-MBaP and BaP included a sub-model of their major phenolic metabolite, 3-OH-8-MBaP and 3-OHBaP, respectively, that was shown able to induce in vitro developmental toxicity in the mEST. The mEST describes the development of pluripotent stem cells into beating cardiomyocytes and quantification of inhibition of this developmental process was previously shown to provide an adequate animal-free testing method for predicting developmental toxicity of petroleum substances and PAHs (Kamelia et al. 2017, 2020). The approach of predicting developmental toxicity using PBK model reverse dosimetry with in vitro concentration–response data obtained from the mEST assay was also validated before for other compounds known to cause developmental toxicity (Louisse et al. 2010; Wang et al 2021). Biotransformation of PAHs plays an important role in their subsequent developmental toxicity (Carrillo et al. 2022; Fang et al. 2022; Incardona et al. 2006; Kamelia et al. 2020). Previously it was shown that the methyl substitution of BaP shifts oxidative metabolism from the aromatic ring to the side chain (Wang et al. 2022). Notably, in the metabolism of 8-MBaP by rat liver microsomes, the metabolic efficiency in formation of 8-hydroxymethyl-BaP (8-OH-8MBaP) was 5.3-fold higher than the formation of the aromatic ring oxidation metabolites. In spite of the extensive conversion of 8-MBaP to 8-OH-8MBaP by side-chain hydroxylation, the metabolic efficiency (defined at V_max_/K_m_) for formation of 3-OH-8-MBaP from 8-MBaP was 5.9 µl/min/mg protein and comparable to that of the conversion of BaP to 3-OHBaP being 7.8 µl/min/mg protein (Wang et al. 2022).

The validation of the developed PBK model for 8-MBaP in rats was achieved by comparing the predicted blood concentrations of 8-MBaP and 3-OH-8MBaP with those that were observed in an in vivo rat experiment with 8-MBaP and BaP and to data available in literature for BaP and 3-OHBaP (Marie et al. 2010; Moreau and Bouchard 2015). The comparable metabolic efficiency for conversion of the parent PAH to its 3-OH metabolite shown in the in vitro kinetic studies are in line with the limited differences between the predicted and observed in vivo blood concentration versus time curves for 3-OH-8-MBaP and 3-OHBaP upon dosing an equimolar amount of the parent PAH, resulting in a similar C_max_. The overall oxidative metabolism of 8-MBaP compared to BaP as a result of the side chain hydroxylation results in faster metabolic clearance resulting in a relatively lower C_max_ of 8-MBaP than for BaP at 10 mg/kg dose with the ratio of C_max_ 8-MBaP/C_max_ BaP being 0.95, 0.48, and 0.77 upon intravenous, intratracheal, and oral exposure, respectively. The fact that this difference was not clearly detected in the in vivo kinetic experiment might be caused by large experimental variation due to the limited number of rats and the fact that recovery of BaP and 8-MBaP from blood samples may be influenced by their high tendency for lipid and protein binding, thus adding variability to the obtained data as well. This would be in line with the deviation observed between in vivo data reported in other in vivo studies as illustrated by the data presented in Fig. 5 for blood concentrations from different studies at similar dose levels. Nevertheless, in line with the predictions, the in vivo experiment performed in the present study shows that the blood concentration of 3-OH-8-MBaP was comparable with that of 3-OHBaP.

The level of uncertainty can be defined by comparison of predictions to available literature data and these deviations appeared to be within 2-fold, generally taken as adequate model performance (Peters and Dolgos 2019; van Tongeren et al. 2022; Punt et al. 2022). This also defines the level of uncertainty in the PBK model predictions. Uncertainties in the outcomes of the QIVIVE are additionally dependent on the uncertainty in the in vivo concentration–response curve and the validity of the mEST for the in vivo situation for rat and human. For rats, this uncertainty can be quantified by comparison of toxicity predictions obtained to available toxicological data. In the present study, the mEST data resulted in predictions of the points of departure (PoD) for developmental toxicity in rats that matched the observed available in vivo data showing that the uncertainty in the toxicodynamics is limited and within the uncertainty factors generally applied in risk assessment of 2.5 for interspecies differences in toxicodynamics.

The validated PBK model for 8-MBaP was used to translate the in vitro concentration–response curve for its 3-OH-8-MBaP metabolite in the mEST into in vivo dose–response predictions for developmental toxicity upon oral exposure of rats to 8-MBaP. Developmental toxicity of 8-MBaP was assumed to be caused by metabolic activation to 3-OH-8-MBaP by cytochrome P450 enzymes following the same pathway as previously elucidated for BaP (Kamelia et al. 2020; Wang et al. 2021). Results of the present study reveal that both 3-OH metabolites affected the differentiation of the ES-D3 cells in the differentiation assay of the mEST with an IC_50_ for 3-OH-8-MBaP that was 1.7-fold lower than that of 3-OHBaP, while parent PAHs BaP and 8-MBaP both tested negative in the mEST.

Translation of the in vitro data for the 3-OH metabolite to an in vivo dose–response curve for the parent PAH allowed quantification of an in vivo ED_50_ for developmental toxicity of 8-MBaP and resulted in a value that was 1.3-fold lower than the ED_50_ for BaP, while in the in vitro mEST, the difference between the IC_50_ values for the related 3-OH metabolites was 1.1-fold. This smaller difference in the predicted IC_50_ values in the mEST than in the in vivo ED_50_ values is related to differences in the kinetics of the two PAHs with the relative conversion to the toxic 3-OH metabolite being somewhat less effective for 8-MBaP than for BaP.

Thus, based on the in vitro mEST data and the predicted in vivo curves for developmental toxicity, the potency of 8-MBaP appears to be, respectively, 1.4- and 1.3-fold higher than that of BaP observed in a previous (Wang et al 2021) and the present study. The higher potency of 8-MBaP as compared to BaP is in line with results of the ZET (Fang et al. 2022) where 8-MBaP appeared also more potent than BaP albeit by a factor 10. This different relative potency of 8-MBaP compared to BaP in the mEST and mEST-based in vivo predictions reported in the present study and the ZET results reported previously may be due to differences in the cytochrome P450 enzymes involved in metabolic activation in the zebrafish embryo’s as compared to the rat liver microsomes and/or in the mode of action underlying the developmental toxicity in zebrafish embryos of the ZET and the mouse ES-D3 cells of the mEST. In addition to the metabolic differences, other factors may also play a role in the differences in toxic potency between the mEST and zebrafish assay, such as species differences in receptor binding affinity, physiological differences, and toxicodynamic susceptibility for the endpoint studied.

The current PBK model predicted the blood concentration and developmental toxicity of 8-MBaP using read-across from the previously developed and validated model for BaP, and quantitative in vitro to in vivo extrapolation (QIVIVE) from in vitro data in the mEST for its 3-OH metabolite. It seems reasonable to assume that the new approach methodology (NAM) that was proven valid for BaP, and uses PBK modeling-facilitated QIVIVE of data from the mEST to predict in vivo dose–response curves for developmental toxicity, will also provide reliable data for the developmental toxicity of the related compound 8-MBaP. Nevertheless, it would be of interest to validate the model and the QIVIVE predictions when in vivo developmental toxicity data in rats would become available in the future. The present study illustrates how NAMs can be applied to predict developmental toxicity of a methyl substituted PAH for which in vivo data may not be available, by PBK modeling facilitated read-across from a non-substituted structural analog for which in vivo data have been reported. The study may, thus, contribute to the need of reducing the use of animal testing for hazard assessment of alkyl-substituted PAHs. In addition, the method elucidates how a methyl substituent could affect the kinetics and subsequent developmental toxicity potency of a non-substituted PAH. The PBK model-facilitated read-across may be of use for further evaluation of other PAHs and methylated PAHs that need bioactivation to induce developmental toxicity. The developed in vitro–in silico approach to predict developmental toxicity of BaP and 8-MBaP in rats can be adapted to prediction for humans by feeding human physiological parameter values and human metabolic kinetic parameters into the PBK model, and using in vitro concentration–response data from human-induced pluripotent stem cells (hiPSCs)-based differentiation assays as input for the QIVIVE. The predicted dose–response curves obtained enable definition of PoDs to define health-based guidance values for safe human exposure and facilitate human risk assessment without the need for data from experimental animals. In future studies, the newly developed PBK models will also facilitate translation of human biomonitoring data for hydroxylated metabolites to the related in vivo dose levels of the corresponding PAHs and the internal concentrations which can subsequently be compared to concentrations needed to trigger an effect in relevant in vitro model systems. Application of NAMs for regulatory purpose needs to be considered with rigorous validation, standardization, and biological relevance for human risk assessment. Consideration of an extra uncertainty factor of 10 to account for uncertainties when using NAMs could overcome this reluctancy. And when a NAM-based prediction of a PoD would be fully based on human PBK and in vitro toxicity models, there would be no need for an uncertainty factor for interspecies differences and an overall uncertainty factor of 100, including a factor of 10 for both inter-individual differences and use of NAMs, would still apply.

## Supplementary Information

Below is the link to the electronic supplementary material.

Supplementary file 1 (DOCX 26 KB)

Supplementary file 2 (DOCX 68 KB)

## Data Availability

The datasets generated and analyzed during the current study are available from the corresponding author upon reasonable request.

## Code availability

Model code is available in Supplementary Material 1.
