# Supplementary material for: Predicting the developmental toxicity of 8-methyl-benzo[a]pyrene (BaP) by physiologically based kinetic (PBK) modeling-facilitated reverse dosimetry and read-across from BaP
Source: Arch Toxicol. 2025 Jul 3;99(10):4035–50. doi: 10.1007/s00204-025-04115-y (PMC12454603; doi:10.1007/s00204-025-04115-y)
Supplement: Supplementary file 1 — (DOCX 26 KB) [file 204_2025_4115_MOESM1_ESM.docx]

;Date:09-06-2022

;Purpose: PBK model 8-methyl-benzo[a]pyrene and 3-hydroxy-8-methyl-benzo[a]pyrene, built with literature, in vitro and in silico derived parameter values

;Species: Rat (gender mixed)

;Compiled by: Danlei Wang, based on PBK models for BaP that included submodels for 3OHBaP

;==========================================================================

;Physiological parameters

;==========================================================================

;tissue volumes (Crowell et al. (2011), based on Brown et al. (1997))

BW = 0.245 {Kg} ; body weight rat (variable, dependent on study)

VFc = 0.065 ; fraction of fat tissue

VLc = 0.037 ; fraction of liver tissue

VLuc= 0.005 ; fraction of lung tissue

VABc =0.0257 ; fraction of arterial blood

VVBc = 0.0514 ; fraction of venous blood

VRc = 0.2159 ; fraction of richly perfused tissue (= 1 - (VFc + VLc + VLuc + VABc + VVBc+ VSc))

VSc = 0.6 ; fraction of slowly perfused tissue

VF = VFc*BW {L or Kg} ; volume of fat tissue (calculated)

VL = VLc*BW {L or Kg} ; volume of liver tissue (calculated)

VLU = VLuc*BW {L or Kg} ; volume of lung tissue (calculated)

VAB = VABc*BW {L or Kg} ; volume of arterial blood (calculated)

VVB = VVBc*BW {L or Kg} ; volume of venous blood (calculated)

VR = VRc*BW {L or Kg} ; volume of richly perfused tissue (calculated)

VS = VSc*BW {L or Kg} ; volume of slowly perfused tissue (calculated)

;--------------------------------------------------------------------------------------------------------------------

;blood flow rates (Crowell et al. (2010), based on Brown et al. (1997))

QC = 15*BW^0.74 {L/hr} ; cardiac output

QFc = 0.07 ; fraction of blood flow to fat

QLc = 0.183 ; fraction of blood flow to liver

QLuc = 1 ; fraction of blood flow to lung

QRc = 0.4 ; fraction of blood flow to richly perfused tissue

QSc = 0.347 ; fraction of blood flow to slowly perfused tissue

QF = QFc*QC {L/hr} ; blood flow to fat tissue (calculated)

QL = QLc*QC {L/hr} ; blood flow to liver tissue (calculated)

QLu = QLuc*QC {L/hr} ; blood flow to lung tissue (calculated)

QS = QSc*QC {L/hr} ; blood flow to slowly perfused tissue (calculated)

QR = QRc*QC {L/hr} ; blood flow to richly perfused tissue (calculated)

;-------------------------------------------------------------------------------------------------------------------

;Intestinal lumen volumes, surfaces, absorption rates, transfer rates

;Papp, Caco-2 = 3.8 {x1E-6 cm/sec} ;in vitro, from Caco-2 cells (Goth-Goldstein et al. (1999)

PappCaco2=-5.42 ; Log Papp, Caco-2

;Log (Papp,in vivo) = 0.6836*Log(PappCaco-2)-0.5579 (Sun et al. 2002)

Papp=10^(0.6836*PappCaco2-0.5579)*3600/10 ; apparent intestinal permeability coefficient in vivo {dm/hr}

; 7-compartment model for GI-tract based on model bisphenol A and 17β-estradiol by Zhang et al., (2018)

Vin = 0.0012 ; volume for each compartment of intestines {L}

SAin = 0.134 ; surface area {dm2}

kin = 4.17 ; transfer rate to next compartment within the intestines {/hr}

;kabin1 = Papp*SAin ; absorption rate constant {L/hr}

Vin1 = Vin ; volume of intestine compartment 1 {L}

SAin1 = SAin ; surface area of intestine compartment 1 {dm2}

kabin1 = Papp*SAin1 ; absorption rate constant of intestine compartment 1 {L/hr}

kin1 = kin ; transfer rate to intestine compartment 2 {/hr}

Vin2 = Vin ; volume of intestine compartment 2 {L}

SAin2 = SAin ; surface area of intestine compartment 2 {dm2}

kabin2 = Papp*SAin2 ; absorption rate constant of intestine compartment 2 {L/hr}

kin2 = kin ; transfer rate to intestine compartment 3 {/hr}

Vin3 = Vin ; volume of intestine compartment 3 {L}

SAin3= SAin ; surface area of intestine compartment 3 {dm2}

kabin3 = Papp*SAin3 ; absorption rate constant of intestine compartment 3 {L/hr}

kin3 = kin ; transfer rate to intestine compartment 4 {/hr}

Vin4 = Vin ; volume of intestine compartment 4 {L}

SAin4 = SAin ; surface area of intestine compartment 4 {dm2}

kabin4 = Papp*SAin4 ; absorption rate constant of intestine compartment 4 {L/hr}

kin4 = kin ; transfer rate to intestine compartment 5 {/hr}

Vin5 = Vin ; volume of intestine compartment 5 {L}

SAin5 = SAin ; surface area of intestine compartment 5 {dm2}

kabin5 = Papp*SAin5 ; absorption rate constant of intestine compartment 5 {L/hr}

kin5 = kin ; transfer rate to intestine compartment 6 {/hr}

Vin6 = Vin ; volume of intestine compartment 6 {L}

SAin6 = SAin ; surface area of intestine compartment 6 {dm2}

kabin6 = Papp*SAin6 ; absorption rate constant of intestine compartment 6 {L/hr}

kin6 = kin ; transfer rate to intestine compartment 7 {hr}

Vin7 = Vin ; volume of intestine compartment 7 {L}

SAin7 = SAin ; surface area of intestine compartment 7 {dm2}

kabin7 = Papp*SAin7 ; absorption rate constant of intestine compartment 7 {L/hr}

kin7 = kin ; transfer rate to co {/hr}

kfe = 0.27 ; transfer rate to faeces {/hr} taken from Crowell et al. (2011)

;=====================================================================

;Physicochemical parameters

;=====================================================================

;partition coefficients 8MBaP (tissue:blood)

PF8MBaP = 434.55 ; fat/blood partition coefficient

PL8MBaP = 12.85 ; liver/blood partition coefficient

PLu8MBaP = 14.65 ; lung/blood partition coefficient

PR8MBaP= 12.85 ; rapidly perfused tissue/blood partition coefficient

PS8MBaP = 7.13 ; slowly perfused tissue/blood partition coefficient

;--------------------------------------------------------------------------------------------------------------------

;partition coefficients 3-OH8MBaP (tissue:blood)

PF3OH8MBaP = 392.83 ; fat/blood partition coefficient of 3-OH8MBaP

PL3OH8MBaP = 12.55 ; liver/blood partition coefficient of 3-OH8MBaP

PLu3OH8MBaP = 14.31 ; lung/blood partition coefficient of 3-OH8MBaP

PR3OH8MBaP = 12.55 ; rapidly perfused tissue/blood partition coefficient of 3-OH8MBaP

PS3OH8MBaP = 6.98 ; slowly perfused tissue/blood partition coefficient of 3-OH8MBaP

;=====================================================================

;Kinetic parameters

;=====================================================================

;Metabolism liver

;MPL: scaling factor of rat liver microsomes (mg microsomal protein /g liver)

MPL=45 ; mg microsomal protein/g liver Reference: (Houston & Galetin (2008))

;Maximum rate of metabolism of 8MBaP to 3-OH8MBaP, measured in vitro in present study

VMax1c= 0.07 {nmol/min/mg microsomal protein} ; 8MBaP -> 3-OH8MBaP

;Maximum rate of metabolism scaled to liver

VMax1 = VMax1c/1000*60*MPL*VL*1000 {µmol/hr/liver}

;Michaelis-Menten constant for metabolism of 8MBaP to 3-OH8MBaP measured in vitro in present study

Km1 = 12 {uM}

;--------------------------------------------------------------------------------------------------------------------

;Maximum rate of metabolism of 8MBaP to remaining metabolites measured in present study

VMax2c = 0.43 {nmol/min/mg microsomal protein} ; (8MBaP-->remaining metabolites)

;Maximum rate of metabolism scaled to liver

VMax2 = VMax2c/1000*60*MPL*VL*1000 {µmol/hr/liver}

;Michaelis-Menten constant for metabolism of 8MBaP to remaining metabolites measured in vitro in present study

Km2 = 8 {uM}

;--------------------------------------------------------------------------------------------------------------------

;Sulfonation of 3-OH8MBaP

;MSL: scaling factor of rat liver S9 (mg S9 protein /g liver)

MSL = 125 ; mg S9 protein/g liver (Houston and Galetin, 2008)

;Maximum rate of metabolism for sulfation of 3-OH8MBaP determined in vitro in present study

VMax3c= 0.15 {nmol/min/mg S9 protein}

VMax3 = VMax3c/1000*60*MSL*VL*1000 {µmol/hr/liver}

;Michaelis-Menten constant for sulfation of 3-OH8MBaP determined in vitro in present study

Km3 = 26 {µM}

;--------------------------------------------------------------------------------------------------------------------

;Glucuronidation of 3-OH8MBaP

;Maximum rate of metabolism glucuronidation of 3-OH8MBaP determined in vitro in present study

Vmax4c= 6.92 {nmol/min/mg S9 protein} ;

Vmax4 = Vmax4c/1000*60*MSL*VL*1000 {µmol/hr/liver}

; Michaelis-Menten constant for glucuronidation of 3-OHBaP determined in vitro

Km4 = 16 {µM}

;=====================================================================

;Run settings

;=====================================================================

;Exposure parameters

;Molecular weight

MW8MBaP = 266.34 ; Molecular weight 8MBaP

MW3OH8MBaP = 282.34 ; Molecular weight 3-OH8MBaP

;IV dose = given IV dose in mg/kg bw

IVDOSEmg = 0 {mg/kg bw}

IVDOSEumol2 = IVDOSEmg*1E-3/MW8MBaP*1E6 {µmol/ kg bw}

;IVDOSEumol2 = given iv dose recalculated to µmol/kg bw

IVDOSEumol=IVDOSEumol2*BW {µmol} ; intravenous dose

;oral dose = given oral dose in mg/kg

ODOSEmg = 0 {mg/kg bw}

ODOSEumol2 = ODOSEmg*1E-3/ MW8MBAP*1E6 {µmol/kg bw}

ODOSEumol=ODOSEumol2*BW; {µmol} ; oral dose

;intratracheal dose = given intratracheal dose in mg/kg

ITDOSEmg = 0 {mg/kg bw}

ITDOSEumol2 = ITDOSEmg*1E-3/MW8MBaP*1E6 {µmol/ kg bw}

;ITDOSEumol = given intratracheal dose recalculated to µmol/kg bw

ITDOSEumol=ITDOSEumol2*BW {µmol} ; intratracheal dose

;Time

Starttime = 0 ; in hr

Stoptime = 24 ; in hr

;=====================================================================

;Model calculations

;=====================================================================

;Exposure routes

Frequency = 24 {h} ; duration between doses, in hours

Repetitions = 1 ; total number of daily doses

;Intravenous, needle

;ANe = amount 8MBaP in needle

ANe' = -kd*ANe

Init ANe = IVDOSEumol

kd=1000000 {/h} ;kd, the transport rate from needle to blood

; intratracheal

;ATr = amount 8MBaP in trachea

ATr' = -kt*ATr

Init ATr = ITDOSEumol

kt = 1 {h} ; absorption from trachea to lung, maximum value assumed

;--------------------------------------------------------------------------------------------------------------------

;Stomach compartment

;Ast = amount of BaP remaining in stomach, µmol

Ast' = -ka*Ast

Init Ast = ODOSEumol

ka = 1 {h} ; Absorption constant stomach to GI-tract maximum value assumed

;--------------------------------------------------------------------------------------------------------------------

;intestinal compartment, divided in 7 sub compartments

;Ain1 = Amount 8MBaP in intestine compartment 1 (µmol)

Cin1 = Ain1/Vin1

Ain1' = ka*Ast - kin1*Ain1 - kabin1*Cin1

Init Ain1 = 0

;Ain2 = Amount 8MBaP in intestine compartment 2 (µmol)

Cin2 = Ain2/Vin2

Ain2' = kin1*Ain1 - kin2*Ain2 - kabin2*Cin2

Init Ain2 = 0

;Ain3 = Amount 8MBaP in intestine compartment 3 (µmol)

Cin3 = Ain3/Vin3

Ain3' = kin2*Ain2 - kin3*Ain3 - kabin3*Cin3

Init Ain3 = 0

;Ain4 = Amount 8MBaP in intestine compartment 4 (µmol)

Cin4 = Ain4/Vin4

Ain4' = kin3*Ain3 - kin4*Ain4 - kabin4*Cin4

Init Ain4 = 0

;Ain5 = Amount 8MBaP in intestine compartment 5 (µmol)

Cin5 = Ain5/Vin5

Ain5' = kin4*Ain4 - kin5*Ain5 - kabin5*Cin5

Init Ain5 = 0

;Ain6= Amount 8MBaP in intestine compartment 6 (µmol)

Cin6 = Ain6/Vin6

Ain6' = kin5*Ain5 - kin6*Ain6 - kabin6*Cin6

Init Ain6 = 0

;Ain7= Amount 8MBaP in intestine compartment 7 (µmol)

Cin7 = Ain7/Vin7

Ain7' = kin6*Ain6 - kin7*Ain7 - kabin7*Cin7

Init Ain7 = 0

;Aco = Amount 8MBaP in colon (µmol)

Aco' = kin7*Ain7- kfe*Aco

Init Aco = 0

ACco' = kin7*Ain7

Init ACco = 0 ; cumulative amount reaching colon

;--------------------------------------------------------------------------------------------------------------------

;feces

;AFA = amount 8MBaP in feces (µmol)

AFe' = kfe*Aco + Kb*AL8MBAP

Init AFe = 0

Kb = 1 ; excretion constant liver to faeces via bile maximum value assumed

;--------------------------------------------------------------------------------------------------------------------

;liver compartment

;AL8MBAP = Amount of 8MBaP in liver tissue (µmol)

AL8MBAP' = kabin1*Cin1 + kabin2*Cin2 + kabin3*Cin3 + kabin4*Cin4 + kabin5*Cin5 + kabin6*Cin6 + kabin7*Cin7 + QL*( CAB8MBAP - CVL8MBAP) - AMMO'- AMMT1' - Kb*AL8MBAP

Init AL8MBAP = 0

CL8MBAP = AL8MBAP/VL

CVL8MBAP = CL8MBAP/PL8MBAP

;AMMO = amount of 8-methyl-BaP metabolized to metabolite 3-OH-8-methyl-Benzo[a]pyrene

AMMO' =VMax1*CVL8MBAP/(Km1 + CVL8MBAP)

init AMMO = 0

;AMMT1 = amount of 8MBaP metabolized to other metabolites

AMMT1' = Vmax2*CVL8MBAP/(Km2 + CVL8MBAP)

init AMMT1 = 0

;--------------------------------------------------------------------------------------------------------------------

;fat compartment

;AF8MBAP = Amount of 8MBaP in fat tissue (µmol)

AF8MBAP' = QF*( CAB8MBAP -CVF8MBAP)

Init AF8MBAP = 0

CF8MBAP = AF8MBAP/VF

CVF8MBAP = CF8MBAP/PF8MBAP

;--------------------------------------------------------------------------------------------------------------------

;tissue compartment richly perfused tissue

;AR8MBAP = Amount of 8MBaP in richly perfused tissue (µmol)

AR8MBAP' = QR*( CAB8MBAP -CVR8MBAP)

Init AR8MBAP = 0

CR8MBAP = AR8MBAP/VR

CVR8MBAP = CR8MBAP/PR8MBAP

;--------------------------------------------------------------------------------------------------------------------

;tissue compartment slowly perfused tissue

;AS8MBAP = Amount of 8MBaP in slowly perfused tissue (µmol)

AS8MBAP' = QS*( CAB8MBAP -CVS8MBAP)

Init AS8MBAP = 0

CS8MBAP = AS8MBAP/VS

CVS8MBAP = CS8MBAP/PS8MBAP

;--------------------------------------------------------------------

;lung blood compartment

;ALU8MBAP = Amount of 8MBaP in lung blood, µmol

ALU8MBAP' = kt*ATr+ QLU*(CVB8MBAP - CVLU8MBAP)

Init ALU8MBAP = 0

CLU8MBAP = ALU8MBAP/VLU

CVLU8MBAP = CLU8MBAP/PLU8MBAP

;--------------------------------------------------------------------

; venous blood

;AVB8MBAP = amount of 8MBaP in venous blood, µmol

AVB8MBAP' = Ane*kd + (QF*CVF8MBaP + QL*CVL8MBaP + QS*CVS8MBaP + QR*CVR8MBaP) - QLU*CVB8MBAP

Init AVB8MBAP = 0

CVB8MBAP = AVB8MBAP/VVB

AUCVB8MBAP' = CVB8MBAP

init AUCVB8MBAP = 0

;--------------------------------------------------------------------

; arterial blood

;AAB8MBAP = amount of 8MBaP in arterial blood, µmol

AAB8MBAP' = QLU*(CVLU8MBAP -CAB8MBAP)

Init AAB8MBAP = 0

CAB8MBAP = AAB8MBAP/VAB

AUCAB8MBAP' = CAB8MBAP

init AUCAB8MBAP = 0

CB8MBaPtot =CVB8MBAP + CAB8MBAP

;========================================================================

;3-hydroxy-8-methyl-benzo[a]pyrene submodel

;========================================================================

;feces

;AFA = amount 3OH8MBaP in feces (µmol)

A3OH8MFe' = kfe*Aco + Kc*AL3OH8MBAP

Init A3OH8MFe = 0

Kc = 1 ;excretion constant liver to faeces via bile maximum value assumed

;--------------------------------------------------------------------------------------------------------------------

;liver compartment

;AL3OH8MBAP = Amount of 3-OH8MBaP in liver tissue (µmol)

AL3OH8MBAP' = AMMO' + QL*( CAB3OH8MBAP - CVL3OH8MBAP) - AMMT2' - AMMT3' - Kc*AL3OH8MBAP

Init AL3OH8MBAP = 0

CL3OH8MBAP = AL3OH8MBAP/VL

CVL3OH8MBAP = CL3OH8MBAP/PL3OH8MBAP

;AMMT2 = Amount of 3-OH8MBaP sulfonated

AMMT2' = Vmax3*CVL3OH8MBAP/(Km3 + CVL3OH8MBAP)

Init AMMT2 = 0

;AMMT3 = Amount of 3-OH8MBaP glucuronidated

AMMT3' = Vmax4*CVL3OH8MBAP/(Km4 + CVL3OH8MBAP)

Init AMMT3 = 0

;--------------------------------------------------------------------------------------------------------------------

;fat compartment

;AF3OH8MBAP = Amount of 3-OH8MBaP in fat tissue (µmol)

AF3OH8MBAP' = QF*( CAB3OH8MBAP -CVF3OH8MBAP)

Init AF3OH8MBAP = 0

CF3OH8MBAP = AF3OH8MBAP/VF

CVF3OH8MBAP = CF3OH8MBAP/PF3OH8MBAP

;--------------------------------------------------------------------------------------------------------------------

;tissue compartment richly perfused tissue

;AR3OH8MBAP = Amount of 3-OH8MBaP in richly perfused tissue (µmol)

AR3OH8MBAP' = QR*( CAB3OH8MBAP -CVR3OH8MBAP)

Init AR3OH8MBAP = 0

CR3OH8MBAP = AR3OH8MBAP/VR

CVR3OH8MBAP = CR3OH8MBAP/PR3OH8MBAP

;--------------------------------------------------------------------------------------------------------------------

;tissue compartment slowly perfused tissue

;AS3OHBAP = Amount of 3-OH8MBaP in slowly perfused tissue (µmol)

AS3OH8MBAP' = QS*( CAB3OH8MBAP -CVS3OH8MBAP)

Init AS3OH8MBAP = 0

CS3OH8MBAP = AS3OH8MBAP/VS

CVS3OH8MBAP = CS3OH8MBAP/PS3OH8MBAP

;--------------------------------------------------------------------

;lung compartment

;ALU3OH8MBAP = Amount of 3-OH8MBaP in lung tissue, µmol

ALU3OH8MBAP' = QLU*(CVB3OH8MBAP -CVLU3OH8MBAP)

Init ALU3OH8MBAP = 0

CLU3OH8MBAP = ALU3OH8MBAP/VLU

CVLU3OH8MBAP = CLU3OH8MBAP/PLU3OH8MBAP

;--------------------------------------------------------------------

; venous blood

;AVB3OH8MBAP = amount of 3-OH8MBaP in venous blood, µmol

AVB3OH8MBAP' = ( (QF*CVF3OH8MBAP + QL*CVL3OH8MBAP + QS*CVS3OH8MBAP + QR*CVR3OH8MBAP) - QLU*CVB3OH8MBAP)*fub3OH8MBAP

Init AVB3OH8MBAP = 0

CVB3OH8MBAP = AVB3OH8MBAP/VVB

AUCVB3OH8MBAP' = CVB3OH8MBAP

init AUCVB3OH8MBAP = 0

fub3OH8MBAP = 0.004 ; 3-OH8MBaP fraction unbound in blood

;--------------------------------------------------------------------

; venous blood protein

;AVBP3OH8MBAP = amount of 3-OH8MBaP in bound to venous blood protein, µmol

AVBP3OH8MBAP' = ( (QF*CVF3OH8MBAP + QL*CVL3OH8MBAP + QS*CVS3OH8MBAP + QR*CVR3OH8MBAP) - QLU*CVB3OH8MBAP)*fb3OH8MBAP

Init AVBP3OH8MBAP = 0

CVBP3OH8MBAP = AVBP3OH8MBAP/VVB

AUCVBP3OH8MBAP' = CVBP3OH8MBAP

init AUCVBP3OH8MBAP = 0

fb3OH8MBaP = 1-fub3OH8MBAP ; 3-OH8MBaP fraction bound to blood protein

;--------------------------------------------------------------------

; arterial blood

;AAB3OH8MBAP = amount of 3-OH8MBaP in arterial blood, µmol

AAB3OH8MBAP' = (QLU*(CVLU3OH8MBAP -CAB3OH8MBAP)) * fub3OH8MBAP

Init AAB3OH8MBAP = 0

CAB3OH8MBAP = AAB3OH8MBAP/VAB

AUCAB3OH8MBAP' = CAB3OH8MBAP

init AUCAB3OH8MBAP = 0

;--------------------------------------------------------------------

; arterial blood protein

;AABP3OH8MBAP = amount of 3-OH8MBaP bound to arterial blood protein, µmol

AABP3OH8MBAP' = (QLU*(CVLU3OH8MBAP - CAB3OH8MBAP))* fb3OH8MBAP

Init AABP3OH8MBAP = 0

CABP3OH8MBAP = AABP3OH8MBAP/VAB

CB3OH8MBaPtot = CVB3OH8MBAP + CVBP3OH8MBAP + CAB3OH8MBAP + CABP3OH8MBAP

;========================================================================

;Mass balance calculations for 8-methyl-Benzo[a]pyrene

Total8MBAP = IVDOSEumol + ITDOSEumol + ODOSEumol

Calculated8MBAP = Ain1 + Ain2 + Ain3 + Ain4 + Ain5 + Ain6 + Ain7 + Aco + AFe + AL8MBAP + AF8MBAP +AS8MBAP + AR8MBAP + AAB8MBAP + AVB8MBAP +ALU8MBAP + AMMO + AMMT1 + ANe + Ast + ATr

ERROR8MBAP=(Total8MBAP-Calculated8MBAP)/(Total8MBAP+1E-30)*100

MASSBBAL8MBAP=Total8MBAP-Calculated8MBAP + 1

;========================================================================

;Mass balance calculations for 3-hydroxy-8-methyl-benzo[a]pyrene sub-model

Total3OH8MBAP = AMMO

Calculated3OH8MBAP = A3OH8MFe + AL3OH8MBAP + AF3OH8MBAP +AS3OH8MBAP + AR3OH8MBAP + AAB3OH8MBAP + AABP3OH8MBAP+AVB3OH8MBAP + AVBP3OH8MBAP + ALU3OH8MBAP + AMMT2 + AMMT3

ERROR3OH8MBAP=(Total3OH8MBAP-Calculated3OH8MBAP)/(Total3OH8MBAP+1E-30)*100

MASSBBAL3OH8MBAP=Total3OH8MBAP-Calculated3OH8MBAP + 1

;========================================================================
