# Supplementary material for: Predicting the developmental toxicity of 8-methyl-benzo[a]pyrene (BaP) by physiologically based kinetic (PBK) modeling-facilitated reverse dosimetry and read-across from BaP
Source: Arch Toxicol. 2025 Jul 3;99(10):4035–50. doi: 10.1007/s00204-025-04115-y (PMC12454603; doi:10.1007/s00204-025-04115-y)
Supplement: Supplementary file 2 — (DOCX 68 KB) [file 204_2025_4115_MOESM2_ESM.docx]

Figure 1. The zoom-in Figure 6 for time dependent blood concentrations with x axis from 0-5 h a) 8-MBaP (red curves) and BaP (black curves) and of b) 3-OH-8-MBaP (red curves) and 3-OH-BaP (black curves) upon intravenous exposure of rats to 50 mg/kg bw 8-MBaP or BaP. For comparison, the measured in vivo blood concentrations in time of a) 8-MBaP (red triangles) and BaP (black squares) and of b) 3-OH-8-MBaP (red triangles) and 3-OHBaP (black squares) are presented.
